# Arterial properties in adults with long-lasting active juvenile idiopathic arthritis compared to healthy controls

**DOI:** 10.1186/s12969-018-0302-5

**Published:** 2018-12-29

**Authors:** Hanne Aaserud Aulie, Mette-Elise Estensen, Anne Marit Selvaag, Vibke Lilleby, Berit Flatø, Svend Aakhus

**Affiliations:** 1 Department of Rheumatology, Oslo University Hospital, Rikshospitalet, Oslo, Norway; 2 Department of Internal Medicine, Diakonhjemmet Hospital, Oslo, Norway; 3 Department of Cardiology, Oslo University Hospital, Rikshospitalet, Oslo, Norway; 4 Institute for Clinical Medicine, Medical Faculty, University of Oslo, Oslo, Norway; 5 Department of Circulation and Imaging, Faculty of Medicine and Health Science, Norwegian University of Science and Technology, NTNU, Trondheim, Norway; 6 Clinic of Cardiology, St. Olavs Hospital, Trondheim, Norway

**Keywords:** Juvenile idiopathic arthritis, Inflammation, Cardiovascular disease, Arterial stiffness

## Abstract

**Background:**

The data on cardiovascular risk and systemic arterial properties in patients with long-lasting juvenile idiopathic arthritis (JIA) is limited. The objective of this study was to describe systemic arterial properties including characteristic impedance (Z_0_), total arterial compliance (C), and peripheral vascular resistance (R) in patients with long-lasting active JIA compared with matched controls, and to assess the relation to JIA disease variables and traditional cardiovascular risk factors.

**Findings:**

*Methods:* Eighty-one JIA patients (median age 38.6) with at least 15 years of active disease were reexamined after median 29 years of disease duration and compared to 41 healthy controls. With use of echocardiography and calibrated right common carotid artery tonometric pulse traces, noninvasive estimates of pressure and blood flow from the aortic root were obtained and used to estimate the systemic arterial parameters Z_0_, C and R.

*Results:* The patients had higher Z_0_ as assessed by Windkessel model (mean ± SD 65.0 ± 30.1 versus 53.4 ± 18.8 10^− 3^ mmHg/ml/s, *p* = 0.027), lower C as assessed by either Windkessel model or ratio of stroke volume and pulse pressure (1.57 ± 0.46 versus 1.80 ± 0.65 ml/mmHg, *p* = 0.030, 1.29 ± 0.37 versus 1.43 ± 0.34 ml/mmHg, *p* = 0.038), and similar R compared to the controls. Years on daily prednisolone and insulin resistance were the most important correlates of Z_0._ Metotrexat use, polyarticular disease course and erythrocyte sedimentation rate were also associated with a higher Z_0._

**Conclusion:**

Our results indicate that JIA patients had altered arterial properties as compared to controls. Years on daily prednisolone and insulin resistance were the most important correlates of altered arterial properties.

## Introduction

Juvenile idiopathic arthritis (JIA) is a chronic inflammatory rheumatic disease with symptom onset in childhood and persists into adulthood in about 50% of the patients [1, 2].

Arterial stiffness is an established independent predictor of cardiovascular disease (CVD) [3]. Whereas adult onset inflammatory arthritis is known to be related to increased arterial stiffness [4], little is known of systemic arterial properties and cardiovascular risk in those with long-lasting JIA.

Pulse wave velocity (PWV) is the most frequently used method for the measurement of arterial stiffness reflecting large arterial stiffness [5]. However, the properties of the arterial system can be described in more detail by use of system level parameters, as characteristic impedance (Z_0_), arterial compliance (C) and vascular resistance (R) [6].

We have previously reported an increased prevalence of hypertension and arterial stiffness as measured by PWV in adults with long-lasting active JIA [7]. However, Z_0_, C and R have hitherto not been described in this patient group. The present study was therefore designed to characterize systemic arterial properties in detail in JIA patients as compared to matched controls. We also wanted to assess the associations between JIA disease variables, cardiovascular risk factors and arterial properties.

## Methods

### Patients and controls

Totally 134 JIA patients with clinically active disease for at least 15 years of disease duration were invited to a combined rheumatologic and cardiologic follow-up study after median 29 years. The patients were initially referred to the Oslo University hospital between 1980 and 85, and re-examined after median 15 and 23 years before the present study [2]. Between May 2011 and March 2012, 90 patients were included in the study. Nine were excluded after inclusion due to pregnancy (*n* = 3), recordings of suboptimal quality (*n* = 5), or severe heart disease without presumed relation to JIA (*n* = 1). The 81 participants were not different from the 53 eligible but not participating patients regarding gender, disease duration and age (data not shown).

Forty-six healthy, age- and gender-matched controls were selected randomly from the Norwegian population register. Responders with a history of diabetes mellitus, hypertension, or inflammatory arthritis were not included. Five were excluded after inclusion due to suboptimal recordings.

### Clinical examination and cardiovascular risk assessments

The 81 patients underwent a clinical examination performed by a specialist in rheumatology, the controls were clinically examined by a physician.

JIA was classified according to the International League of Associations for Rheumatology criteria [8]. Active disease was defined as the lack of remission off anti-rheumatic medication [9].

Family history of CVD was defined as a first degree having CVD before the age of 65 (women) and 55 (men). Information about smoking habits and physical activity was obtained through a questionnaire. Insulin resistance was derived from the assessments of insulin and glucose [10].

Three measurements of systolic blood pressure (SBP) and diastolic blood pressure (DBP) with a difference of < 5 mmHg were averaged and taken in all participants after 5 min rest in a supine position. Arterial hypertension was defined as SBP > 140 mmHg and/or DBP > 90 mmHg, and/or use of antihypertensive medication.

Blood samples were drawn after an overnight fast and analysed for high sensitive C-reactive protein (CRP), erythrocyte sedimentation rate (ESR), insulin, glucose and cholesterol.

### Non-invasive assessment of arterial properties

#### Tonometry

We recorded the arterial pulse waveform from the right common carotid artery with the patient in a supine position, using a high-fidelity external applanation tonometric device (Millar SPT-301, Millar Instruments Inc., Houston, USA). Recordings were obtained semi-simultaneously with echocardiographic doppler recordings (Vivid 7 ultrasound scanner, GE Vingmed ultrasound, Norway) of blood flow from the left ventricular outflow tract and the parasternal short axis midventricular cineloops. The tonometric signal was amplified and transferred to a personal computer for processing in Matlab 7 application [11]. We selected at least three cardiac cycles for analysis. The carotid pulse trace peak and nadir were then calibrated with the systolic and diastolic brachial arterial pressures, respectively.

#### Systemic arterial properties

A 3-element electrical analogue Windkessel model of the systemic circulation was used to assess Z_0_, R and C [11]. In order to obtain estimates of systemic parameters independent of any assumed model, we also assessed Z_0_ in the frequency domain as the average of the high frequency harmonics of the input impedance modulus [6]. C was also estimated as stroke volume (mL) over arterial pulse pressure (mmHg).

#### Statistics

The JIA patients and controls, and the patient subgroups were compared using a 2-sided independent sample *t*-test for the continuous normally distributed variables, the Mann-Whitney *U*-test for the continuous not normally distributed values, and the χ^2^ test for the categorical variables. For the continuous normally distributed variables, central tendencies were given as the mean ± SD and for the continuous not normally distributed values, median inter-quartile range. A *p* value < 0.05 (2 tailed) were regarded as statistically significant for all the analyses. Pearson’s Correlation coefficients and multivariate linear regression analysis were used to investigate associations between disease variables, cardiovascular risk factors and arterial properties. SPSS Version 20 (SPSS, Chicago, USA) was used for the statistical analyses.

## Results

### Demographics and cardiovascular risk factors

The characteristics of patients and controls are summarized in Table 1. Hypertension was present in 11% of the patients, and in none of the controls (*p* = 0.028).

**Table 1:** JIA disease characteristics and traditional cardiovascular risk factors

Variables	JIA patients (*n* = 81)	Controls (*n* = 41)
Demographics		
Male gender; n (%)	20 (25)	9 (22)
Age (years); median (IQR)	38.6 (34.9–40.7)	37.7 (34.8–40.5)
Disease duration (years); median (IQR)	29.3 (28.3–30.6)	
Onset age (years); median (IQR)	8.9 (4.9–11.8)	
JIA subtype distribution		
Systemic arthritis; n (%)	4 (5)	
RF negative polyarthritis; n (%)	11 (14)	
RF positive polyarthritis; n (%)	5 (6)	
Persistent oligoarthritis; n (%)	14 (17)	
Extended oligoarthritis; n (%)	13 (16)	
Entesitis related arthritis; n (%)	18 (22)	
Psoriatic arthritis; n (%)	14 (17)	
Unclassified arthritis; n (%)	2 (3)	
Current medication at 29-year follow-up		
Anti-TNF; n (%)	25 (31)	
Metotrexat; n (%)	19 (24)	
NSAIDs daily; n (%)	23 (28)	
Prednisolone; n (%)	5 (6.2)	
Cardiovascular risk factors		
BMI (kg/m^2^); mean (SD)	25.7 (5.0)	25.3 (3.9)
Waist circumference (cm); mean (SD)	92.7 (12.7)	92.7 (9.0)
Daily smokers; n (%)	18 (22)	5 (12)
CVD in first degree relative; n (%)	46 (57)	19 (46)
Hypertension; n (%)	9 (11)	0 (0)*
Myocardial infarction; n (%)	1 (1)	0 (0)
Total cholesterol (mmol/L); mean (SD)	4.9 (1.1)	4.9 (0.8)
LDL cholesterol (mmol/L); mean (SD)	3.0 (1.0)	3.0 (0.8)
hs-CRP (mg/L); median (IQR)	1.8 (0.7–5.0)	0.7 (0.0–1.9)*
Insulin resistance	1.1 (1.1)	0.9 (0.6)

*JIA* Juvenile idiopathic arthritis, *IQR* inter-quartile range, *RF* rheumatoid factor, anti-*TNF* anti-tumor necrosis factor, *NSAIDs* nonsteroidal anti-inflammatory drugs, *BMI* body mass index, *LDL* low density lipoprotein, *CVD* cardiovascular disease, *hs-CRP* high sensitivity C-reactive protein

^*^ *p* < 0.05 compared with values in previous column

### Systemic arterial properties

Z_0_ estimated by Windkessel model was significantly higher (21.7%, *p* = 0.027, Table 2) in the patients, and Z_0_ by frequency domain analyses although numerically higher in the patients, the difference was not statistically different between the groups. C by either of Windkessel model or ratio of stroke volume and pulse pressure was significantly lower in the patients compared to controls (12.8 and 9.8%, *p* = 0.030 and *p* = 0.038, respectively). Heart rate was slightly higher in the patients (*p* = 0.026), however R was similar in patients and controls.

**Table 2:** Arterial haemodynamics and arterial properties

Variables	JIA Patients (*n* = 81)	Controls (*n* = 41)	Difference (%)	*P*-value
Height, cm	170.5 (8.4)	170.3 (8.3)	0	0.881
Weight, kg	74.9 (16.7)	73.3 (12.1)	2.2	0.603
Heart rate, beats/minute	65 (11)	61 (9)	6.6	0.026
Systolic blood pressure, mmHg	119 (18)	113 (11)	5.3	0.054
Diastolic blood pressure, mmHg	70 (10)	67 (9)	4.5	0.091
Mean arterial pressure; mmHg	90 (13)	85 (9)	5.9	0.026
R, mm Hg/ml/s	1.02 (0.27)	1.00 (0.22)	0.02	0.636
C WK, ml/ mm Hg	1.57 (0.46)	1.80 (0.65)	12.8	0.030
C SV/PP, ml/ mmHg	1.29 (0.37)	1.43 (0.34)	9.8	0.038
Z_0_ WK, 10^−3^ mmHg/ml/s	65.0 (30.1)	53.4 (18.8)	21.7	0.027
Z_0_ FD	96.1 (37.2)	91.5 (31.4)	5.0	0.497

Values are the mean (SD)

*R* total peripheral resistance, *C WK* compliance estimated by Windkessel model, *C SV/PP* compliance estimated as ratio of stroke volume and pulse pressure, *Z*_*0*_ *WK* characteristic impedance estimated by Windkessel model, *Z*_*0*_ *FD* characteristic impedance estimated by frequency domain analyses

### The association between JIA disease variables, cardiovascular risk factors and systemic arterial properties

**Fig. 1:**
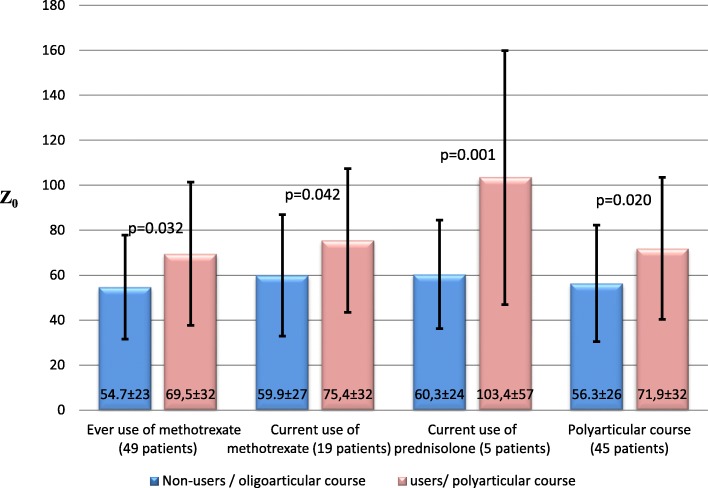
The influence of JIA anti-rheumatic medication and JIA disease burden on systemic arterial properties; legends: JIA = Juvenile idiopathic arthritis, Z_0_ Windkessel model = characteristic impedance estimated by Windkessel model

Z_0_ by Windkessel model was significantly higher in the patients with present or previous use of metotrexat, using prednisolone, and/or had polyarticular disease course as compared to the other patients (Fig. 1), and was correlated to higher ESR area under the curve (AUC), years on daily prednisolone and insulin resistance (Table 3). When including these variables in a multiple linear regression analysis, years on daily prednisolone and insulin resistance were determinants of Z_0_ (ß = 0.003,*p* = 0.021, ß = 0.006,*p* = 0.042).

**Table 3:** Correlations of JIA disease variables, traditional cardiovascular risk factors and arterial properties assessed by Z_0_ Windkessel model

	R	*P*-value
JIA disease variables		
CRP area under the curve^a^	0.095	0.402
ESR area under the curve^a^	0.238	0.037
Years on daily prednisolone	0.346	0.002
Years on daily NSAIDs	0.152	0.177
Years on daily metotrexat	0.094	0.403
Disease duration	−0.193	0.085
JADAS	−0.084	0.465
Number of active joints	0.035	0.758
Traditional cardiovascular risk factors		
Age	−0.117	0.296
BMI	0.180	0.108
Waist circumference	0.213	0.060
Insulin resistance	0.273	0.015
Daily smoking	0.072	0.526
Vigorous physical activity (hours/ week)	−0.111	0.347
Moderate physical activity (hours/ week)	−0.189	0.118

Variables assessed at 29-year follow-up unless otherwise stated

^a^Calculated from parameters assessed at 15-year and 29-year follow-up (CRP), disease onset, 15-year and 29-year follow-up (ESR)

*JIA* juvenile idiopathic arthritis, *CRP* C-reactive protein, *ESR* erythrocyte sedimentation rate, *NSAIDs* nonsteroidal anti-inflammatory drugs, *JADAS* Juvenile Arthritis Disease Activity Score, *BMI* body mass index

## Findings

The present long-term follow-up study indicates that JIA patients with long-term active disease have altered systemic arterial properties as demonstrated by a higher Z_0_ by Windkessel and a lower C when compared to controls. Years on daily prednisolone and insulin resistance were the most important correlates of Z_0_. Furthermore, the use of metotrexat, polyarticular disease course and ESR AUC correlated with higher Z_0_.

Whereas Z_0_ reflects the combined effect of size and stiffness (a high Z_0_ implies a stiffer and/or small sized aorta) of predominantly the ascending aorta, C reflects the volume compliance of the entire systemic arterial tree. R is mainly determined by the small muscular arterioles [6]. Considering both C and the Z_0_ by Windkessel, our findings suggest that the central conduit arteries are altered more than peripheral arteries in adult JIA patients. Previous reports have emphasized the importance of large arterial stiffness rather than alterations of microvascular resistance in the prediction of cardiovascular risk in patients with hypertension [12, 13]. Importantly, lower C and higher Z_0_ are associated with increased cardiovascular risk in the adult general population [14, 15].

Our data support a previous study from the same patient cohort, showing higher PWV in adult JIA patients compared to controls [7]. Thus, increased arterial stiffness has been demonstrated to be present by different noninvasive techniques in JIA patients.

In the patient group, 8 had hypertension while controls with hypertension were excluded in order to maintain a healthy control group. However, when compared to controls, the differences in SBP and DBP were only marginal numerically, and not of statistical significance. Thus, we do not think that the difference in arterial properties between the JIA patients and controls can be fully explained by blood pressure variations. On the other hand, increased vascular stiffness predicts incident hypertension [16] underscoring the bidirectional relationship between arterial properties and blood pressures. In our previous study not excluding hypertensive controls we found an increased prevalence of hypertension in the JIA patients as compared to the controls [7].

We found an association between use of antirheumatic medication, polyarticular disease course and higher ESR AUC and insulin resistance and altered arterial properties. Larger prospective controlled studies are needed to identify determinants of altered arterial properties in JIA patients. Our findings add to the previously reported association between prednisolone use and increased arterial stiffness in JIA patients from our cohort [7].

The strengths of this study are the inclusion of a well-defined cohort of JIA patients followed for 29 years and the fact that detailed information of arterial properties has not been presented in JIA patients before. However, the number of included participants was relatively small, and because of a cross sectional study design the prognostic value of our findings is unknown.

In conclusion, our results suggest that adult patients with long-lasting active JIA had stiffer proximal aorta, and lower total arterial compliance but similar systemic resistance compared to matched controls from the general population. Years on daily prednisolone and insulin resistance were the most important correlates of altered arterial properties. Whether our findings indicate that JIA patients should enter a regular BP monitoring program needs to be determined in future longitudinal follow-up studies.

## Abbreviations

AUC: Area under the curve
C: Total arterial compliance
CRP: C-reactive protein
CVD: Cardiovascular disease
DBP: Diastolic blood pressure
ESR: Erythrocyte sedimentation rate
JIA: Juvenile idiopathic arthritis
PVW: Pulse wave velocity
R: Peripheral vascular resistance
SBP: Systolic blood pressure
Z_0_: Characteristic impedance
